# Back analysis for stratigraphic structure of earthquake-induced soft-hard interbedded anti-dip Rannai Paleolandslides on the Southeastern Qinghai-Tibet Plateau

**DOI:** 10.1038/s41598-026-45484-3

**Published:** 2026-04-04

**Authors:** Jia Jia, Baopin Wen, Zhongjian Zhang, Heng Zhao

**Affiliations:** 1 China University of Geosciences (Beijing), No 29, Xueyuan Road, Haidian District, Beijing, 100083 China; 2 Beijing University of Technology, No 100, Pingleyuan Road, Chaoyang District, Beijing, 100124 China

**Keywords:** Soft-hard interbedded anti-dip rock slopes, Soft-hard interbedded thickness ratio, Back analysis, Slope failure surfaces, Earthquake, Discrete element method, Natural hazards, Civil engineering

## Abstract

Steep terrain and debris cover pose significant challenges to conventional investigation techniques for characterizing the pre-failure stratigraphic structure of soft-hard interbedded anti-dip rock slopes (SHIADRSs). To overcome this, this study develops a numerical back-analysis methodology to quantitatively determine the soft-hard interbedded thickness ratio (SHIR) for paleolandslides. The methodology integrates high-precision unmanned aerial vehicle remote sensing with the universal distinct element code (UDEC). The core of this approach involves simulating the seismic failure processes of numerical models with varying SHIR values and determining the optimal SHIR by maximizing the geometric fitness between the simulated failure surfaces and the actual landslide morphology. Applied to the Rannai Landslide (RNL) on the southeastern Qinghai-Tibet Plateau, the back-analysis yielded a pre-failure SHIR of 1:2.5 (hard rock dominant). This result shows a discrepancy of less than 5% compared to the local stratigraphic data, robustly validating the accuracy and reliability of the proposed back-analysis method. Furthermore, this study elucidated the failure pattern of the RNL under seismic loading, providing valuable insights into the dynamic instability mechanisms of such slopes.

## Introduction

Earthquake-triggered landslides are among the most destructive geological hazards, often resulting in significant loss of life and substantial property damage^1–3^. Soft-hard interbedded anti-dip rock slopes (SHIADRSs) are particularly prone to large-scale failures under seismic loading due to their distinct lithological composition and structural characteristic. For example, the 2005 Kashmir earthquake induced the Hattian Bala landslide in Pakistan, which involved sandstone-shale interbedded slope with a volume of ~ 100 × 10^6^ m^3^^4^. Similarly, the 2008 Wenchuan earthquake triggered the Chengxi landslide in Beichuan County, a slope with sandstone-slate interbedded slope with a volume of 2 × 10^6^ m^3^^5^. Therefore, understanding the failure mechanisms and conditions of SHIADRSs under seismic loading is critical for early hazard identification, risk assessment, and disaster mitigation design.

The soft-hard interbedded structure, prevalent in sedimentary and metamorphic rock strata, is characterized by irregular thickness distributions of soft and hard rock layers. In engineering geology, given the significant differences in the mechanical properties of hard and soft rocks, researchers have commonly simplified the complex interbedded structure using the soft-hard interbedded thickness ratio (SHIR) to characterize the spatial configuration. Over the past decade, experimental studies using rock-like materials have demonstrated that the SHIR profoundly influences the failure characteristics, strength parameters, and damage evolution of rock mass^6–9^. Furthermore, studies on rock surrounding tunnels and slope stability have confirmed that the SHIR is a critical structural parameter governing instability patterns^10,11^. Recent numerical investigations have further quantified the pivotal control of the SHIR on the stability and failure mechanisms of anti-dip slopes, reinforcing its importance as a key geotechnical parameter^11–14^. Accurate determination of the SHIR is therefore essential for elucidating the mechanical behavior of soft-hard interbedded geological bodies.

Conventional methods for determining the SHIR, such as outcrop scan-line surveys, borehole sampling, and adit investigations, rely on systematic analysis of geological profiles and/or core data to derive regional stratigraphic parameters (Table 1)^13,15–20^. However, for SHIADRSs that have undergone large-scale failure—specifically, for paleolandslides—these methods face significant challenges. The rear scarp of such a slope often exhibits deep, brittle failure surfaces, while the central and toe regions are subject to substantial post-failure modifications, including (1) structural disruption of sliding surfaces due to debris flow erosion and (2) extensive coverage of shear zones by collapsed deposits and loose rock fragments. These factors preclude the accurate determination of the SHIR using traditional techniques. Consequently, developing methods suitable for acquiring stratigraphic structural characteristics of SHIADRSs that have experienced large-scale failure has become an urgent need in slope stability research.

**Table 1:** Cases of SHIR determination using geological investigation methods.

Method	Landslide	Slope structure	SHIR	Volume	References
Outcrop scan-line method	Jiaxi Landslide	Anti-dip slope	9:1	3.3 × 10^6^ m^3^	^15^
	Madaling Landslide	Subhorizontal bedding slope	4:1–10:1	1.90 × 10^6^ m^3^	^17^
Borehole sampling method	Toppling failure at the Cihaxia Hydropower Station	Anti-dip slope	1:1–1:5	Height of 350–400 m and depth of approximately 70–100 m	^18^
	A landslide near Badong in the Three Gorges Reservoir Area	Dip slope	1:1	The failure surface extends for 395 m, and the vertical relief is 142 m	^19^
Adit investigation method	Miaowei Slope	Anti-dip slope	1:1	32 × 10^6^ m^3^	^20^

Previous studies have explored the influence of the SHIR on SHIADRSs failure processes through model tests and numerical simulations^11,15,21^. While these studies, such as the recent work of Guo et al. (2023)^12^, have successfully established a correlation between SHIR and slope failure behavior, they primarily adopted a forward-analysis approach to assess stability or understand failure mechanisms. These studies found that while SHIR variations minimally affect macroscopic failure patterns, they significantly influence the geometry of the failure surface. (1) In hard rock-dominated slopes, failure surfaces form large-aperture polygonal patterns, transitioning from slightly convex to planar or concave as the proportion of hard rock increases and with decreasing failure surface depth. (2) In soft rock-dominated slopes, two failure surfaces often develop, with the primary surface exhibiting a concave, curved morphology and the depth positively correlated with the proportion of soft rock. These findings provide a theoretical foundation for the inverse problem: inferring the unknown stratigraphic structural parameters (SHIR) from the failure surface geometry.

To address this inverse problem and the specific technical challenge of determining pre-failure stratigraphy in paleolandslides, this study develops a novel numerical back-analysis method that integrates unmanned aerial vehicle (UAV) remote sensing imagery and discrete element numerical modeling. In contrast to the forward-analysis paradigm prevalent in the literature, our back-analysis framework is designed to solve for the cause (the SHIR) from the effect (the observed failure morphology). The methodology involves the following: (1) field geological investigation and mechanical parameter determination; (2) pre-failure terrain reconstruction; (3) construction of discrete element numerical models for SHIADRSs with varying SHIR values; (4) simulation of dynamic failure processes under identical seismic loading boundary conditions; and (5) evaluation of the geometric matching degree between the simulated 2-D failure surfaces and field topographic features to determine the optimal SHIR value. The application of this approach to the Rannai Landslide (RNL) demonstrates its capability to quantitatively determine the pre-failure SHIR, offering a new tool for paleolandslide investigation.

## Methods

**Fig. 1:**
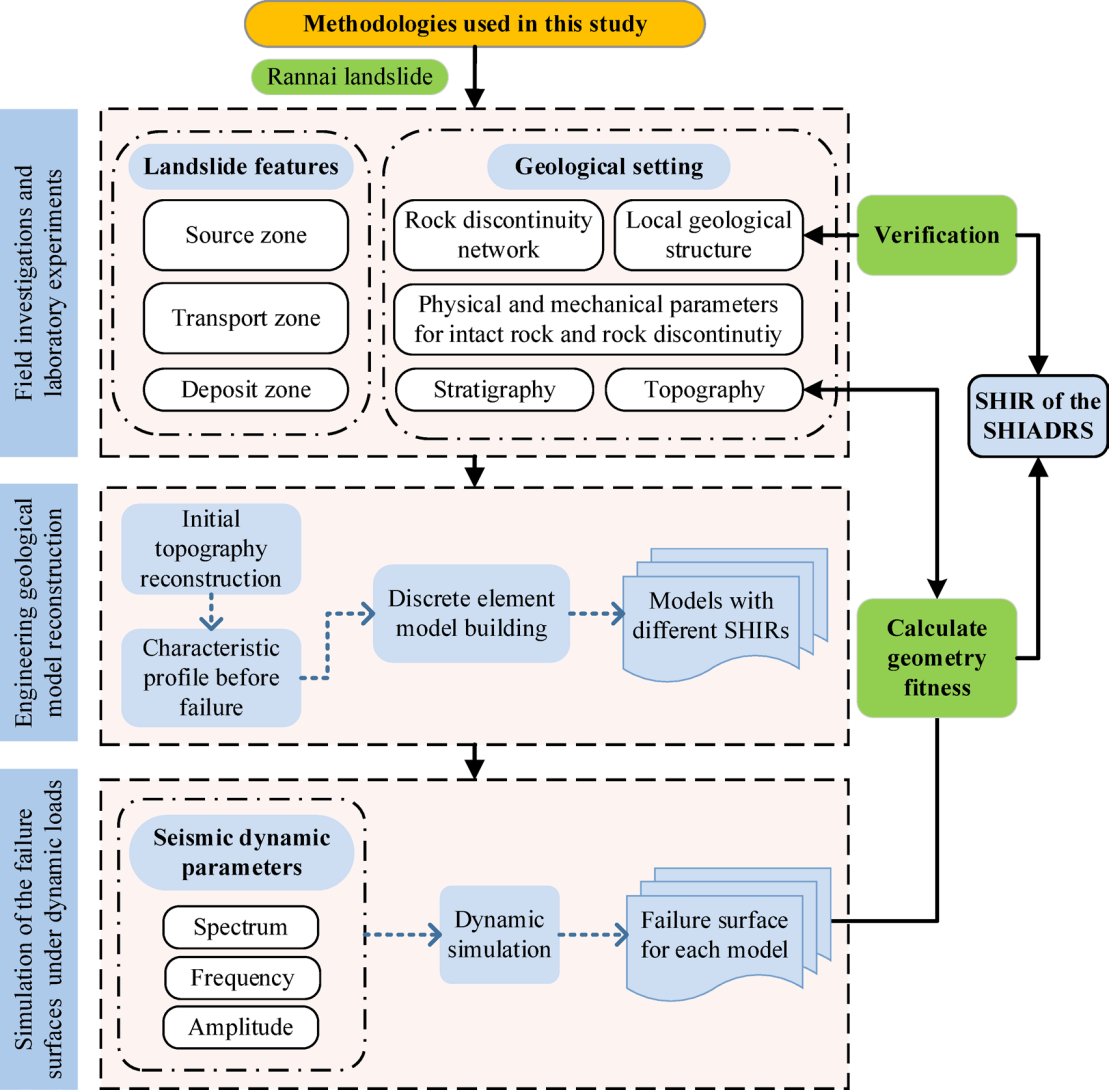
Flow chart of the proposed method.

The methodology developed in this study for back-analysis of SHIR parameters for pre-failure SHIADRSs under seismic loading consists of the key steps outlined below (Fig. 1).

### Field investigation and parameter measurement

Step 1: Geological Background

This step involved identifying the lithology of the strata, measuring the stratigraphic attitude, conducting joint network statistics, and acquiring UAV remote sensing imagery of the landslide area.

Step 2: Landslide characterization

A comprehensive characterization of the landslide features was a prerequisite for conducting numerical back-analysis. The following key features were identified and quantified, each playing a critical role in constraining the numerical model:

- *Pre-Failure Topography Reconstruction*: The volume of the deposit, calculated from detailed morphological mapping of the accumulation area, was used to reconstruct the pre-failure topography of the source area, providing the fundamental geometric basis for all subsequent numerical models.
- *Slope Structural Geometry*: Field measurements of strata attitude were crucial for confirming the anti-dip structure of the slope. This geometric configuration is a first-order control on the potential failure mechanisms and was, therefore, a fundamental input parameter for the model setup.
- *Rock Mass Properties*: The orientation and intensity of discontinuities (e.g., joints), summarized in rose diagrams, informed the definition of the rock mass properties and the potential fracture networks within the UDEC model.
- *Validation Benchmark*: The statistical measurement of the soft-hard layer thickness ratios from exposed outcrops provided the ground-truth data against which the back-analyzed SHIR from the numerical simulations was compared and validated.

Step 3: Laboratory Rock Mechanics Testing

This step focused on determining rock density, uniaxial compressive strength, shear strength, tensile strength, and joint shear strength, thereby providing basic parameters for discrete element numerical simulation.

### Initial terrain reconstruction

The reconstruction of the pre-failure terrain was fundamental, as it provides the essential digital landscape upon which all numerical models are built. This can be achieved by first estimating the volume of the deposit from detailed morphological mapping of the deposit area, which was then used to reconstruct the original geometry of the source area prior to the landslide event.

Step 1: Calculation of Deposit Mass Volume

High-precision UAV remote sensing data were used to construct a 3-D terrain model of the landslide area. The deposit thickness was calibrated based on field investigation results to calculate the deposit mass volume ${V}_{D}$.

Step 2: Back-Calculation of Mass Volume of the Landslide

During the movement process, landslide debris undergoes disintegration and fragmentation. After the movement ceases, the redistribution of the blocks tends toward volume expansion ^22^. Therefore, a volume expansion coefficient $\eta$ was introduced, with a range of 18–35% based on the porosity-particle size relationship model^23^. The specific value of $\eta$ was further determined from the block size distribution of the deposit. Accordingly, the mass volume ${V}_{S}$ of the landslide was back-calculated as follows:

1 $${V}_{S}=\frac{{V}_{D}}{1+\eta }$$

Step 3: Drawing a 2-D Profile Along the Main Sliding Direction

**Fig. 2:**
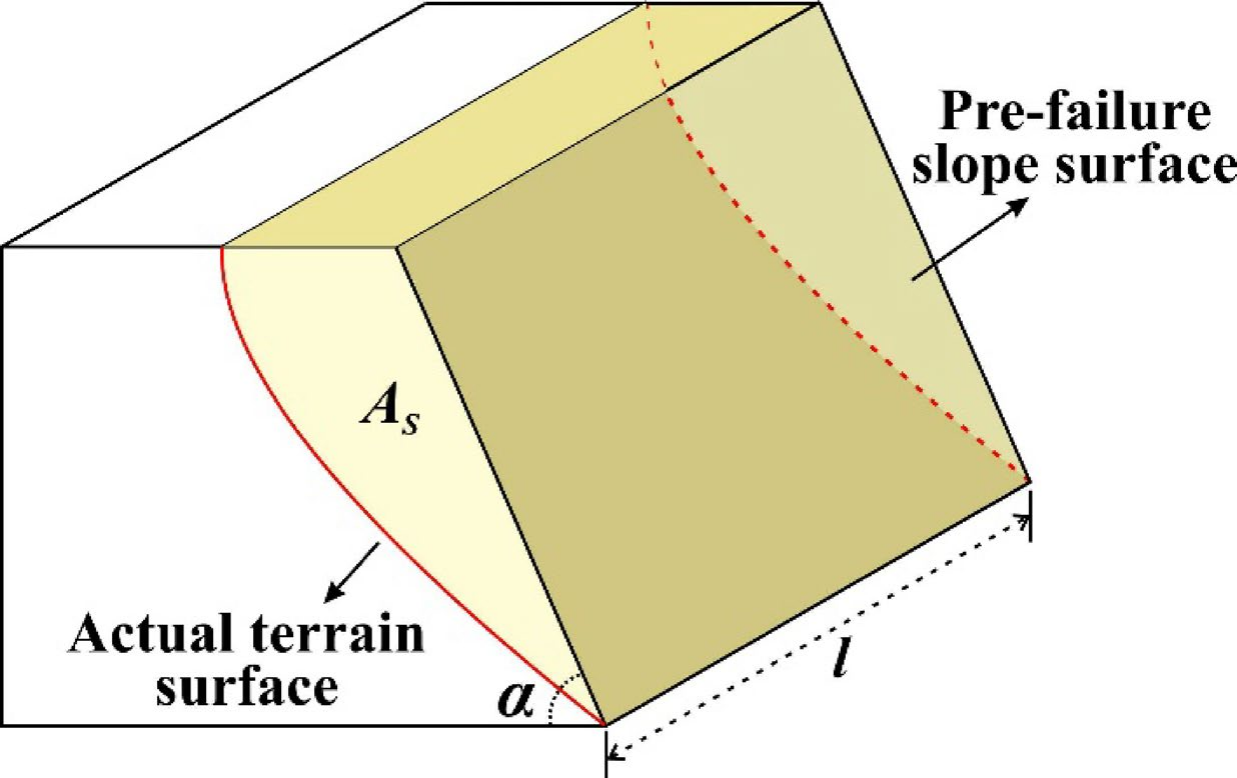
Schematic of the slide volume simplification.

Previous studies have suggested that the failure surface of an SHIADRS can be simplified as an arc surface passing through the slope toe^11^. Therefore, the sliding body was approximated as a triangular prism with a horizontal top surface, a base parallel to the main sliding direction, and an arc-shaped landslide failure surface on one of the sides (Fig. 2).

According to the principle of calculating the volume of a triangular prism, the cross-sectional area ${A}_{S}$ of the slide along the main sliding direction was calculated as follows:

2 $${A}_{S}=\frac{{V}_{S}}{l},$$

where $l$ is the height of the prism, representing the width of the landslide shear exit, which was measured from the 3-D topographic model.

Subsequently, the 2-D terrain profile along the main sliding direction was extracted from the 3-D terrain model. The parameter $\alpha$ (the inclination of the pre-failure slope surface) was iteratively adjusted until the envelope area, bounded by the reconstructed pre-failure slope surface, the slope crest, and the actual post-failure terrain surface, equaled the calculated cross-sectional area ${A}_{S}$. This procedure yielded the final 2-D terrain profile representing the slope geometry prior to failure.

### Geological-numerical model construction

Step 1: Construction of a 2-D Discrete Element Numerical Model

The Universal Distinct Element Code (UDEC)^24^ has been widely applied to analyze the stability and toppling failure mechanisms of anti-dip slopes^25^. However, traditional UDEC models have significant limitations in simulating crack initiation and propagation within rock masses, making it difficult to capture the evolution of failure surfaces in anti-dip slopes. The UDEC Damage Model (UDEC-DM) is a modeling approach based on damage theory^26^. It introduces randomly sized polygonal blocks within rock masses and assigns mechanical parameters matching the rock strength to block boundaries and simultaneously simulates both shear failure along bedding planes and tensile/shear failure within rock blocks in layered rock slopes^27^. This approach has been successfully used to reproduce the geometric characteristics of SHIADRS failure surfaces in model experiments^11,16^. Thus, in this study, the UDEC-DM was employed to construct a 2-D numerical model for simulating the failure surfaces of SHIADRS models with different SHIRs under dynamic loading based on geological profiles of landslides. The rock layer dip angles were derived from the joint network statistics. Randomly sized polygonal blocks were generated within the rock layers using the Voronoi algorithm.

Step 2: Constitutive Relationship and Mechanical Parameters

The Mohr-Coulomb elastoplastic model was adopted as the constitutive relationship for the blocks, while the Coulomb slip model was assigned to the joints. The requisite mechanical parameters were assigned based on the laboratory test results.

Step 3: Construction of SHIADRS Models with Different SHIR Values

Based on the established geological-mechanical model, a series of numerical models were constructed. These models shared consistent dimensions but featured varying SHIR values, achieved by controlling the thicknesses of the soft and hard rock layers. During the model construction, rock layers with very small thickness ratios was treated as interlayers or directly ignored. Consequently, the SHIR was varied across a gradient from 0.25 to 4.0 in this study.

### Dynamic failure process simulation

Step 1: Restoring Initial Stress Field

For large rocky slopes, the initial ground stress field is critical for the accuracy of subsequent dynamic analysis results. The initial stress field was generated through a static analysis under gravitational loading according to elastic theory. The boundary conditions were set as follows: displacement constrained in the x-direction on both sides and displacement constrained in the y-direction at the bottom^28^.

Step 2: Dynamic Loading Parameters

The appropriate selection of dynamic loading parameters is crucial for assessing the dynamic stability of anti-dip slopes^29,30^. In the absence of specific seismic monitoring data for the site, the input vibration was simplified as a sinusoidal wave, mathematically represented as follows:

3 $$V={V}_{max} \cdot \mathit{sin}\left(2 \cdot \pi \cdot f \cdot t\right),$$

where $f$ is the characteristic seismic frequency (Hz). $V$ is the seismic wave velocity (m/s).${V}_{max}$ is the peak wave velocity (m/s) and was calculated as follows:

4 $${V}_{max}=\frac{PGA}{\left(2\cdot \pi \cdot f\right)},$$

where $PGA$ is peak ground acceleration (g).

Additionally, the effective duration $t$ (s) was determined using the statistical relationship established by Bommer et al.^31^ as follows:

5 $$\mathit{log}\left(t\right)=0.69\cdot {M}_{S}-3.70,$$

where ${M}_{s}$ is the seismic magnitude ranging from 5.0 to 8.0. Typically, $f$, $PGA$, and ${M}_{s}$ were estimated based on regional seismic history.

According to the UDEC Manual^24^, the velocity-time history should be converted into normal and shear stress excitations applied at the model boundaries using the following equations:

6 $${\sigma }_{n}=2\left(\rho \cdot {V}_{p}\right)\cdot v,$$

7 $${\sigma }_{s}=2\left(\rho \cdot {V}_{s}\right)\cdot v ,$$

where ${\sigma }_{n}$ and ${\sigma }_{s}$ are the normal and shear stresses, respectively; $\rho$ is mass density; and $v$ is the seismic wave velocity–time history curve. ${V}_{p}$ and ${V}_{s}$ are the P-wave and S-wave velocities within the rock mass, respectively, and was calculated as follows:

8 $${V}_{p}=\sqrt{\left(K+\frac{4}{3}G\right)/\rho }$$

9 $${V}_{s}=\sqrt{G/\rho }$$

where $K$ and $G$ are the bulk and shear moduli of the rock block, respectively, and determined from laboratory tests.

Step 3: Simulating the Dynamic Failure Process

Under identical seismic loading, SHIADRS models with different SHIR values exhibited divergent behaviors, either undergoing dynamic instability or remain stable. In models that failed, a continuous failure surface, typically manifesting as an irregular curve or polyline in the 2-D discrete element model, developed from within the slope mass and propagated to the toe.

### Determination of the SHIR value

Step 1: Quantitative Assessment of Geometric Fitness

**Fig. 3:**
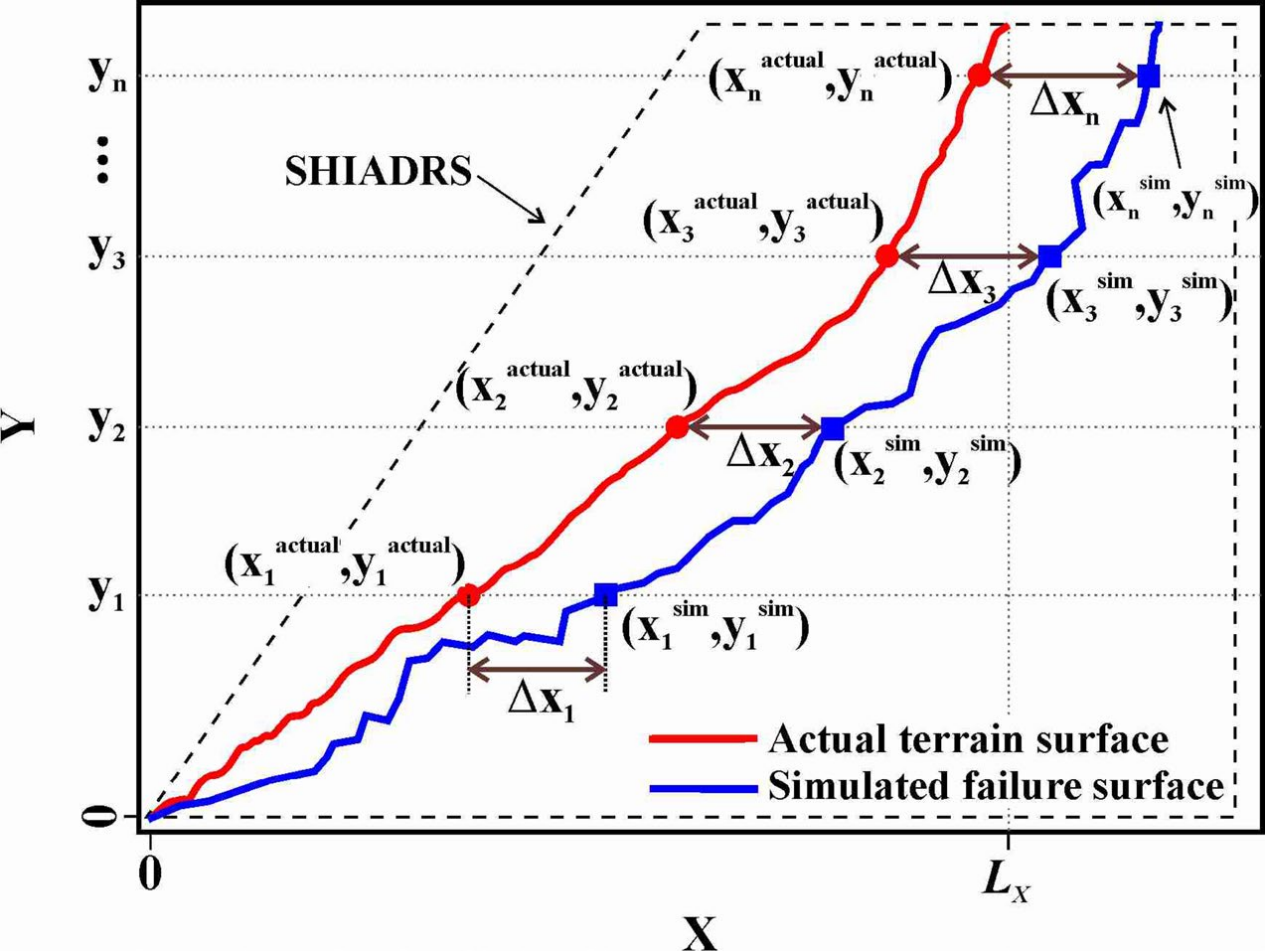
Schematic of the geometric fitness degree calculation principle.

To objectively identify the optimal SHIR, the geometric fitness degree $F$ was introduced as a quantitative parameter to evaluate the match between the simulated failure surface and the actual landslide topography. As illustrated in Fig. 3, $F$ was calculated based on the average absolute deviation along the X-axis. The procedure was conducted as follows: First, for a given SHIADRS model, place the simulated failure surface and actual topography in the same coordinate system with the slope toe as the origin. Second, the slope height was discretized into $N$ uniformly distributed points along the y-axis. The coordinates of the actual topographic curve ${\left.\left({x}_{i}^{actual},{y}_{i}^{actual}\right)\right|}_{i=1}^{N}$ and the simulated failure surface curve ${\left.\left({x}_{i}^{sim},{y}_{i}^{sim}\right)\right|}_{i=1}^{N}$ were recorded for each point $i$. The absolute deviation at each point was calculated as:

10 $$\Delta {x}_{i}=\left|{x}_{i}^{actual}-{x}_{i}^{sim}\right|.$$

Then, the average deviation $\overline{x}$ of $\Delta {x}_{i}$ was computed as follows:

11 $$\overline{x}=\frac{1}{N}{\sum }_{i=1}^{N}\Delta {x}_{i}.$$

Using the X-axis length ${L}_{X}$ of the actual topographic curve as a reference, $F$ was calculated and normalized as follows:

12 $$F=\left(1-\frac{\overline{x}}{{L}_{X}}\right)\times 100\mathrm{\%}.$$

This calculation was performed for all dynamically unstable SHIADRS models, yielding a unique $F$ value for each simulated SHIR scenario.

Step 2: Determination of the Optimal SHIR

The SHIR value that yielded the failure surface with the highest $F$ value was selected as the optimal solution, representing the stratigraphic structure that best replicates the observed landslide morphology. This objective, data-driven selection process directly links the simulation output to the back-analyzed parameter.

Step 3: Validation Against Field Investigation Results

The reliability of the back-analysis was rigorously validated by comparing the optimal SHIR solution with the statistical results of the local outcrop stratigraphic data from field investigations.

## Case study

### Field investigation and parameter measurement

#### Geologic background

**Fig. 4:**
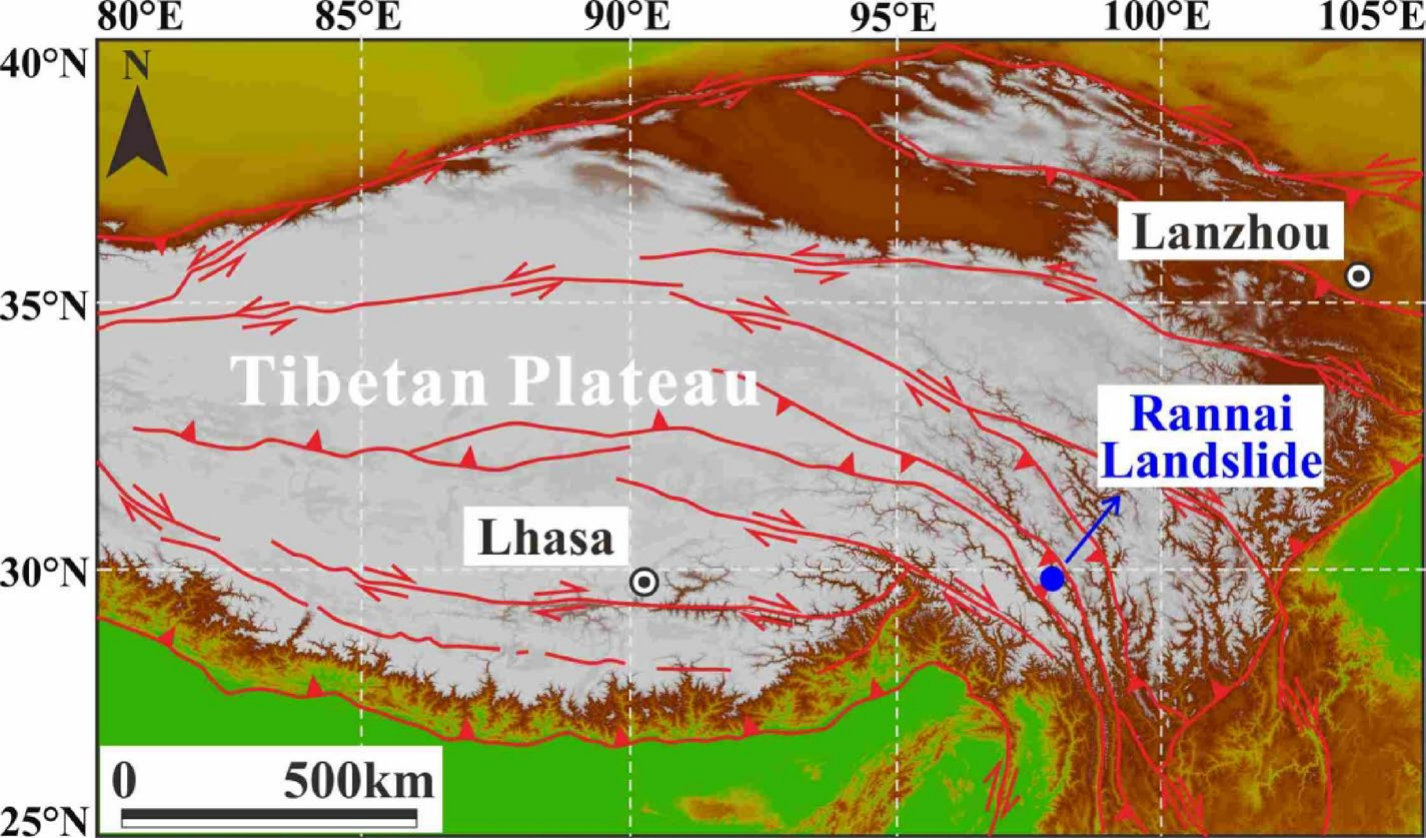
Geologic map showing the tectonic setting of the Qinghai-Tibet Plateau. This map was generated using ArcGIS® software by Esri. ArcMap™ (version 10.8; URL:http://www.esri.com/).

The RNL is located in Zuogong County, Tibet Province, southwestern China, on the southeastern margin of the Qinghai-Tibet Plateau, within the northern segment of the Three Rivers (Nu River, Lancang River, and Jinsha River) region. This area lies at the junction of the Indian and Eurasian plates and is the region in China with the greatest topographic gradient. According to the regional geological framework of the Qinghai-Tibet Plateau (Fig. 4), the RNL is situated between the Bangong Lake-Nu River suture zone and the Lancang River suture zone, and intense tectonic activity occurs within and around these suture zones^32,33^. Previous studies have determined that earthquakes are a key trigger of slope instability in this region^34,35^. Thus, we hypothesize that the RNL was also seismically induced, which has been confirmed by subsequent numerical simulations.

#### Landslide characteristics

**Fig. 5:**
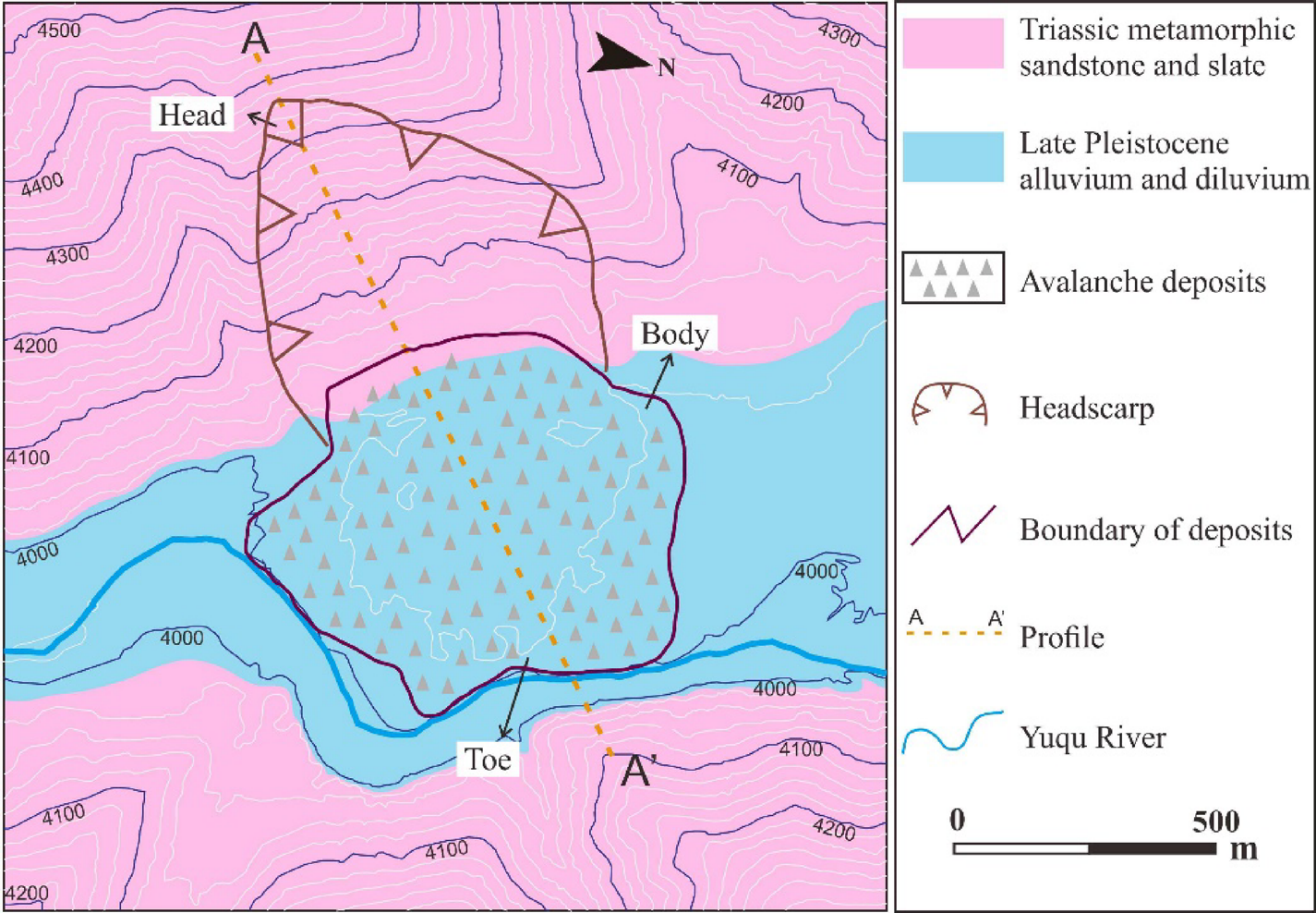
Engineering geological map of the RNL.

High-precision topographic mapping (0.50 m resolution) of the landslide area was conducted using UAV remote sensing. The deposit thickness was measured in the field using a TruPulse200X laser rangefinder, and representative rock samples were collected. The engineering geological map of the RNL is shown in Fig. 5.

**Fig. 6:**
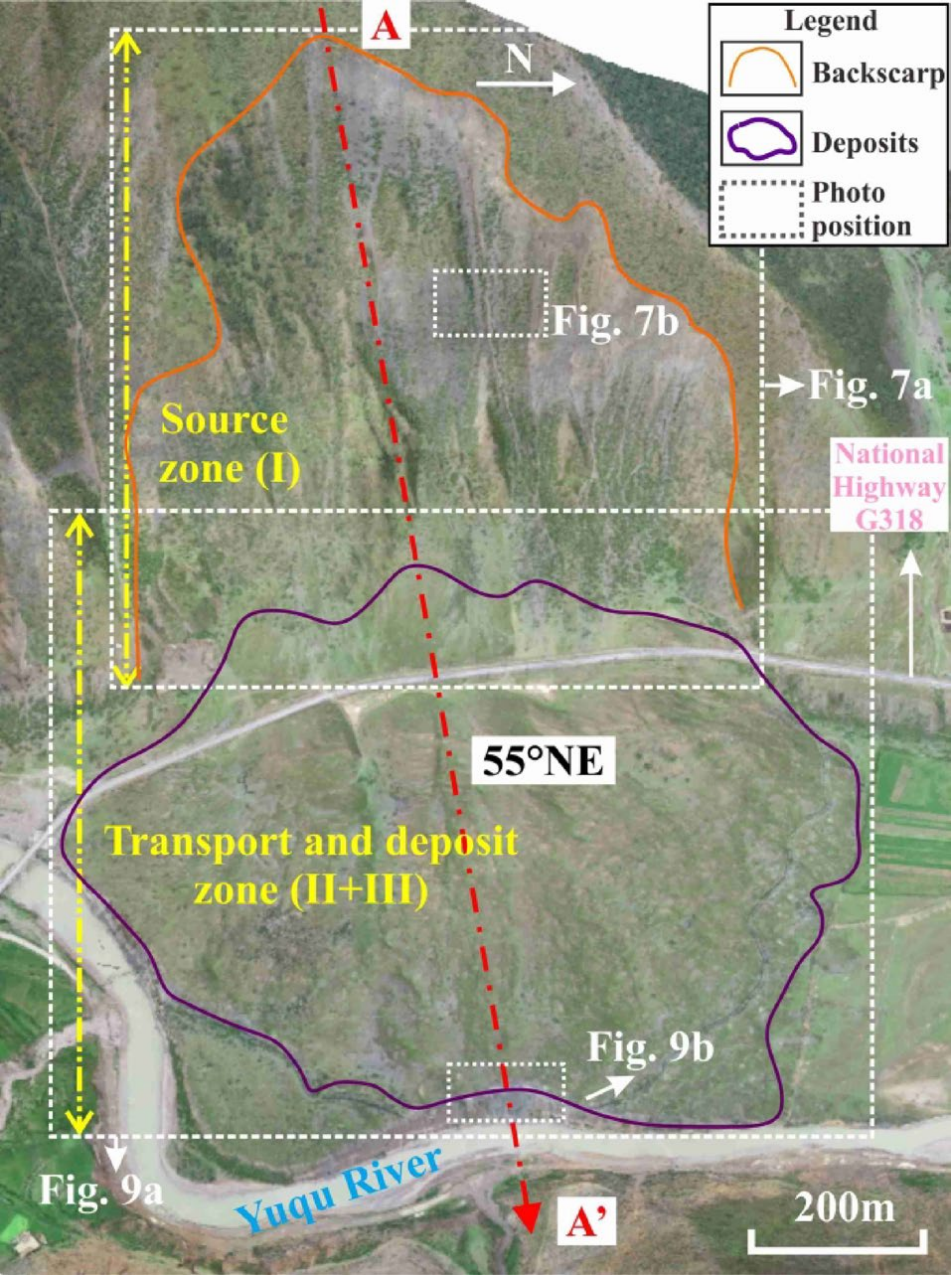
Aerial photograph showing a panoramic view of the RNL. The base image was captured from Google Earth Pro (version 7.3.6.9345; URL:https://www.google.com/earth/) in July 2008. Annotations and geological interpretations were added using CorelDRAW Graphics Suite (version X7; URL:https://www.coreldraw.com/).

Figure 6 presents an aerial photograph of the RNL, revealing that it is a tongue-shaped landslide in plan view. After detaching from the source zone, the landslide mass moved downslope and spread in a lava-like manner across the valley. The movement path was unobstructed, following a straight trajectory with a main sliding direction of 55°NE. The deposit filled the entire width of the valley, obstructed the Yuqu River, leading to the formation of a landslide-dammed lake and ultimately causing river channel diversion. Additionally, based on the following field observations: (1) the severe weathering of the rocks in the source area; (2) the large thickness of vegetation cover on the deposit’s surface; and (3) the large thickness of lake-phase sediments on the in the ground of the landslide-dammed lake area currently, it is hypothesised that the RNL is an ancient landslide.

Based on the surface morphology, the landslide was divided into two primary zones: the source zone and the transport and deposition zone.

**Fig. 7:**
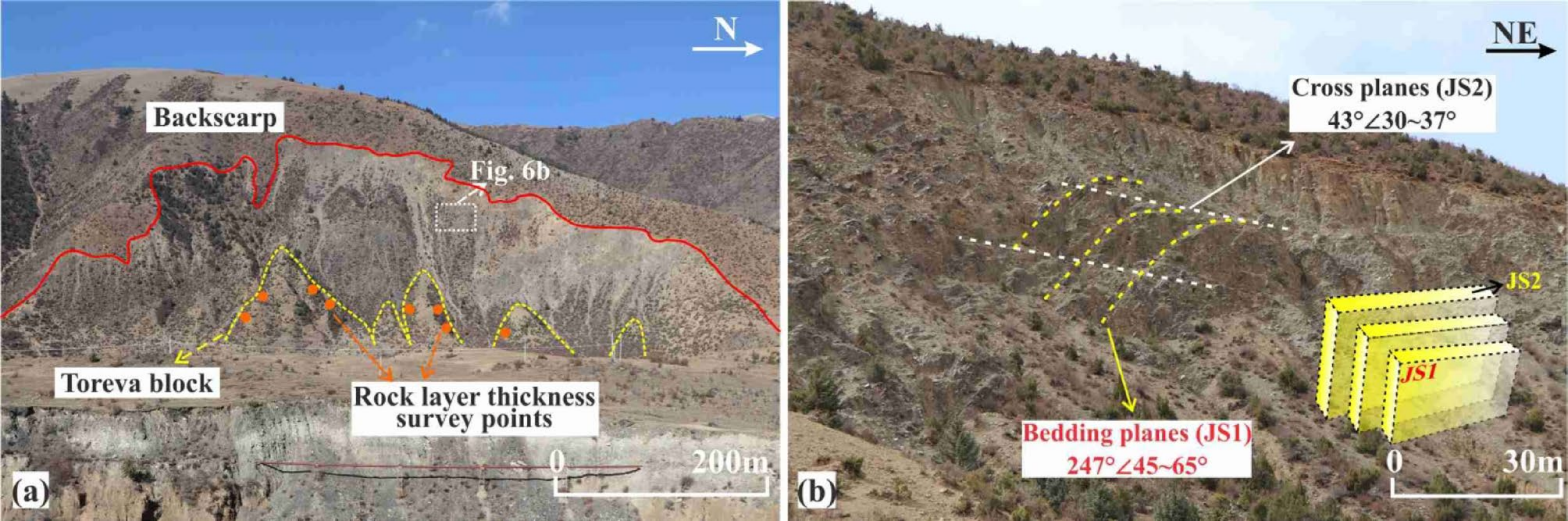
Source zone and two dominant joint sets: (**a**) trailing edge and Toreca blocks; (**b**) bedding planes and orthogonal joints.

**Fig. 8:**
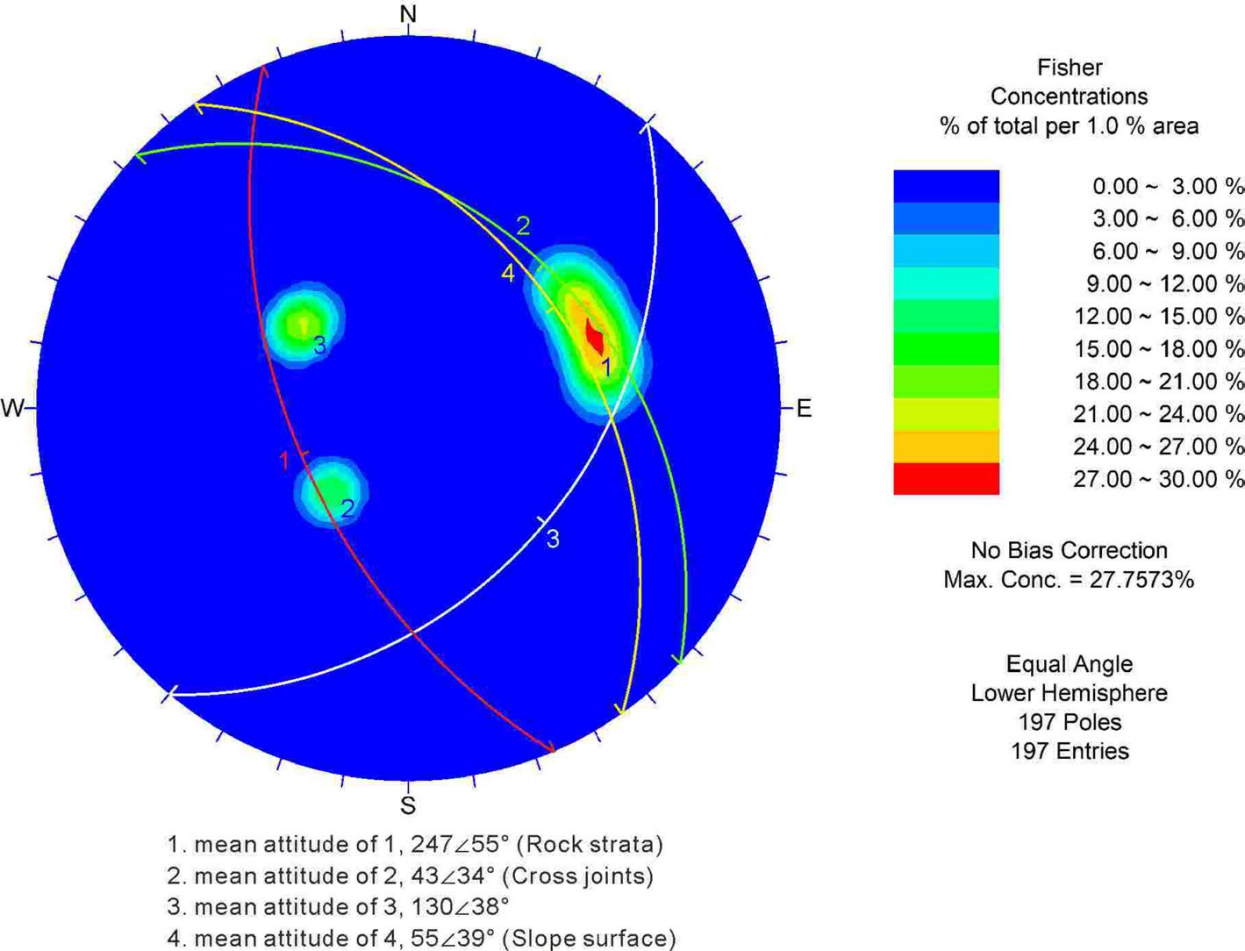
Stereographic projection of dominant structural planes and the slope surface in the source zone.

*Source Zone (Zone I)*: The entire source zone is armchair-shaped in plan view, with a maximum width of approximately 512 m and elevations ranging from 4100 to 4360 m (Fig. 7a). The middle and lower parts are covered by rock fragments of varying sizes. The rear edge is nearly vertical, and the exposed rock layers exhibit characteristics of brittle fracture (Fig. 7b). The landslide stratigraphy comprises metamorphic sandstone and slate. A scan-line survey of structural planes conducted in the central gully, yielding 197 joint measurements. Statistical analysis of these data (Fig. 8) identified two dominant sets of structural planes (JS1 and JS2). JS1 consists of continuous bedding planes dipping into the slope with dip angles of 45–65°. JS2 consists of discontinuous joints dipping out of the slope, with dip angles of 30–37°. A secondary joint set (JS3) dips out of the slope, with angles of 35–45°. Based on the spatial relationship between the bedding plane (JS1) and the pre-failure slope surface, the slope is classified as a typical anti-dip slope.

**Fig. 9:**
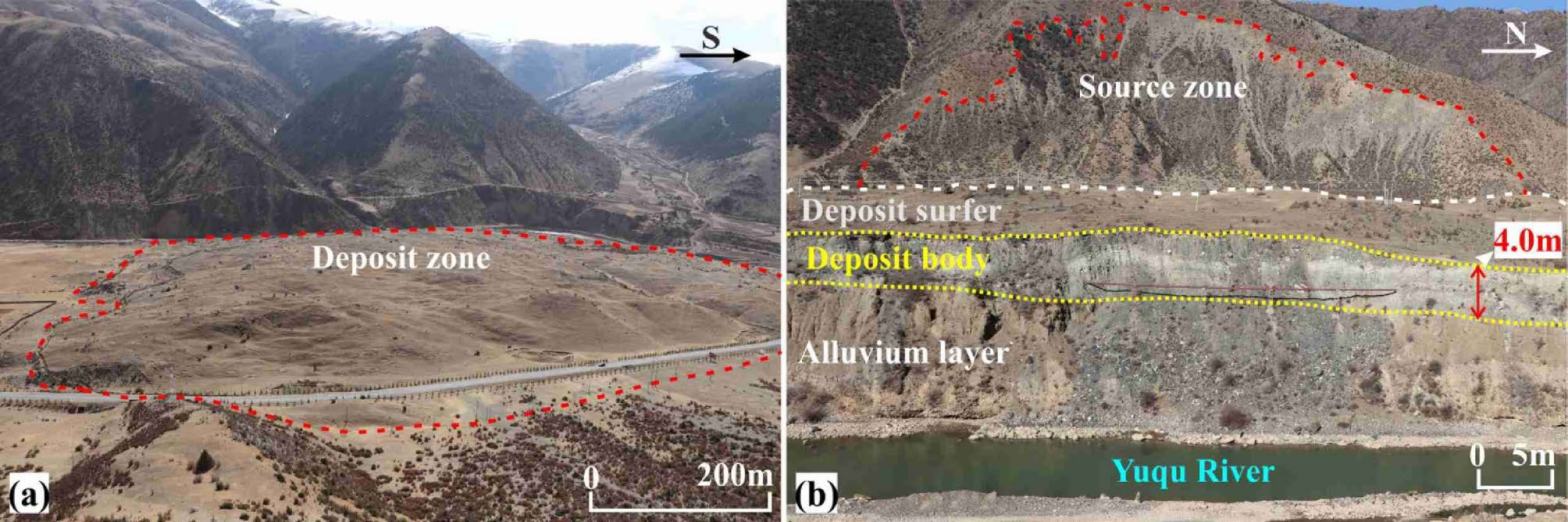
Transport and deposition zone characteristics: (**a**) overview of the zone; (**b**) geological section of the front edge.

*Transport and Deposition Zone (Zones II and III)*: This zone exhibits a shell-like shape in plan view (Fig. 9a), with elevations of 4000–4050 m, a maximum width of approximately 860 m, and a vertical projection area of approximately 4.4 × 10^5^ m^2^. National Highway G318 crosses the boundary between the source and deposit zones. The frontal edge of the deposit has been eroded by the diverted Yuqu River, exposing a longitudinal profile where the deposits interface with fluvial and alluvial sediments (Fig. 9b). Assuming the original valley was planar, the maximum deposit thickness was estimated to be 40 m at the rear of the deposit zone, and an average thickness of approximately 29 m.

#### Mechanical parameters

**Fig. 10:**
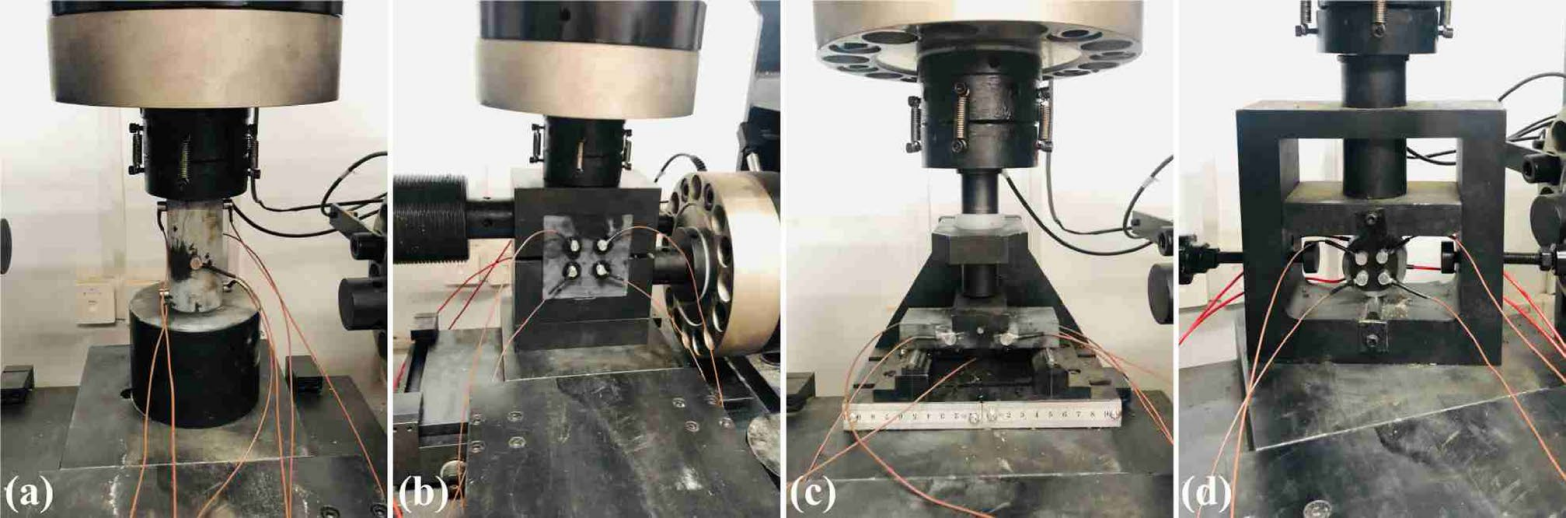
Rock mechanics tests: (**a**) uniaxial compression test; (**b**) direct shear test; (**c**) three-point bending test; (**d**) Brazilian splitting test.

Rock mechanical tests were conducted in accordance with the International Society for Rock Mechanics (ISRM) suggested methods. A YZW-50 universal testing machine was employed to measure the following parameters of rock samples: elasticity modulus, Poisson’s ratio, uniaxial compression strength, shear strength, flexural strength, and tensile strength. A representative sample during testing is shown in Fig. 10. The physical and mechanical parameters presented in Table 2 are the mean ± one standard deviation, calculated from all valid tests for each rock type.

**Table 2:** Physical and mechanical parameters of rocks obtained from laboratory testing.

Parameter	Symbol	Unit	Metamorphic sandstone value (mean ± SD)	Slate value (mean ± SD)	Sample Size (N)
Unit weight	*γ*	kN/m^3^	27.3 ± 0.5	25.7 ± 0.6	12
Elasticity modulus	E	GPa	146.0 ± 12.0	98.0 ± 9.0	
Poisson’s ratio	*μ*		0.30 ± 0.03	0.14 ± 0.02	
Uniaxial compressive strength	*σ*_c_	MPa	180 ± 15	45 ± 6	
Tensile strength	*σ*_t_	MPa	6.0 ± 0.8	0.4 ± 0.1	8
Internal friction angle	*φ*	°	44 ± 3	22 ± 2	10
Cohesion	*c*	MPa	8.0 ± 1.0	3.0 ± 0.5	

The rock mass characteristic mechanical parameters were assessed using modified Geological Strength Index (GSI)^36^. Based on field assessments, GSI values of 80 and 60 were assigned to the metamorphic sandstone and slate, respectively. The strength parameters of the rock mass were then calculated using the generalized Hoek–Brown failure criterion^37^ and are presented in Table 3.

**Table 3:** Mechanical parameters of rock masses.

Rock Mass	Elasticity modulus E (GPa)	Poisson’s ratio *μ*	Uniaxial compressive strength *σ*_c_ (MPa)	Tensile strength *σ*_t_ (MPa)	Internal friction angle *φ* (°)	Cohesion *c* (MPa)
Metamorphic sandstone	128	0.30	60	1.9	39	6.0
Slate	51	0.14	4.8	0.1	18	0.9

In the UDEC-DM SHIADRS model, mechanical parameters were assigned to three distinct joint types: (1) polygonal mesh joints representing the internal defects within the metamorphic sandstone; (2) polygonal mesh joints representing the internal defects within the slate; and (3) bedding plane joints. The mechanical parameters of the first two types of joints were derived directly from Table 3. The parameters for the bedding planes were defined with reference to previous research^16,38^ and empirical judgment. All joint mechanical parameters used in the simulation are summarized in Table 4.

**Table 4:** Mechanical parameters of joints.

Joint	Tensile strength *σ*_t_ (MPa)	Internal friction angle *φ* (°)	Cohesion *c* (MPa)	Normal stiffness (GPa·m^-1^)	Shear stiffness (GPa·m^-1^)
Polygonal mesh of metamorphic sandstone	1.9	39	6.0	46.0	40.0
Polygonal mesh of slate	0.1	18	0.9	12.0	10.0
Bedding plane	0	8	0.004	16.0	14.0

### Initial topography reconstruction

#### Volume calculation

**Fig. 11:**
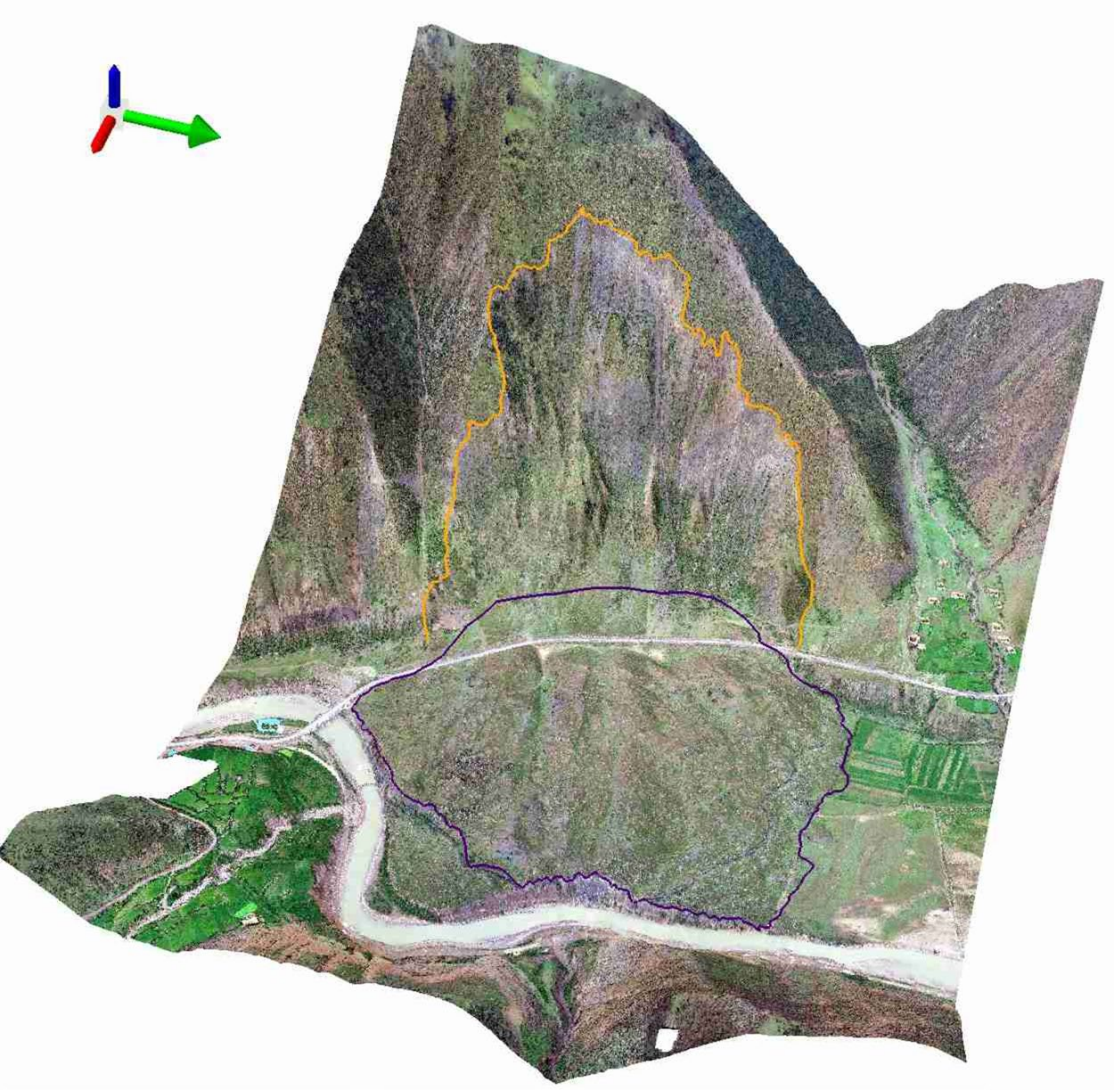
Three-dimensional topographic modeling of the RNL.

A high-precision 3-D terrain model of the RNL (Fig. 11) was constructed using the ArcScene software^39^, an ArcGIS 3D Analyst extension, based on the UAV remote sensing data. Through measurements and calculations, it was determined that ${V}_{D}$ ≈ 12.8 × 10^6^ m^3^ and $l$ = 323 m.

Assuming that $\eta$ was 26% for the mass of the RNL and using Eq. (1), ${V}_{S}$ was calculated to be 10.2 × 10^6^ m^3^.

#### Profile along the main sliding direction

**Fig. 12:**
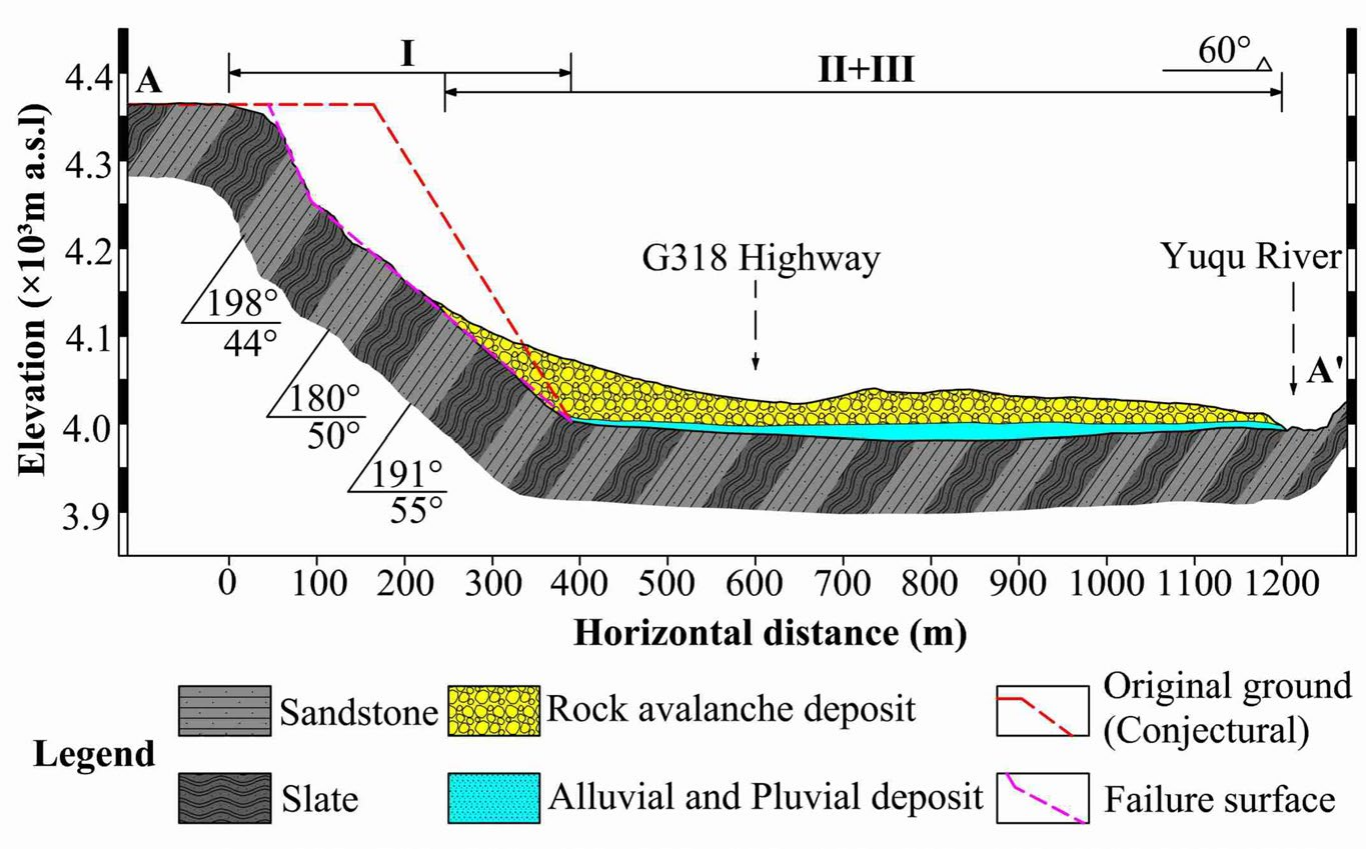
Geological profile of RNL along cross-section A-A’ (Fig. 6).

Substituting ${V}_{S}$ into Eq. (2) yielded ${A}_{S}$. was 3.15 × 10^4^ m^2^. The 2-D terrain profile was then extracted along the main sliding direction (line A-A' in Fig. 6) from the 3-D terrain model. Through an iterative procedure, $\alpha$ was determined to be approximately 58°. Based on these parameters, the pre-failure geological profile of RNL along the main sliding direction was reconstructed, as shown in Fig. 12.

### Construction of geological-numerical model

#### Construction of the basic discrete element model

**Fig. 13:**
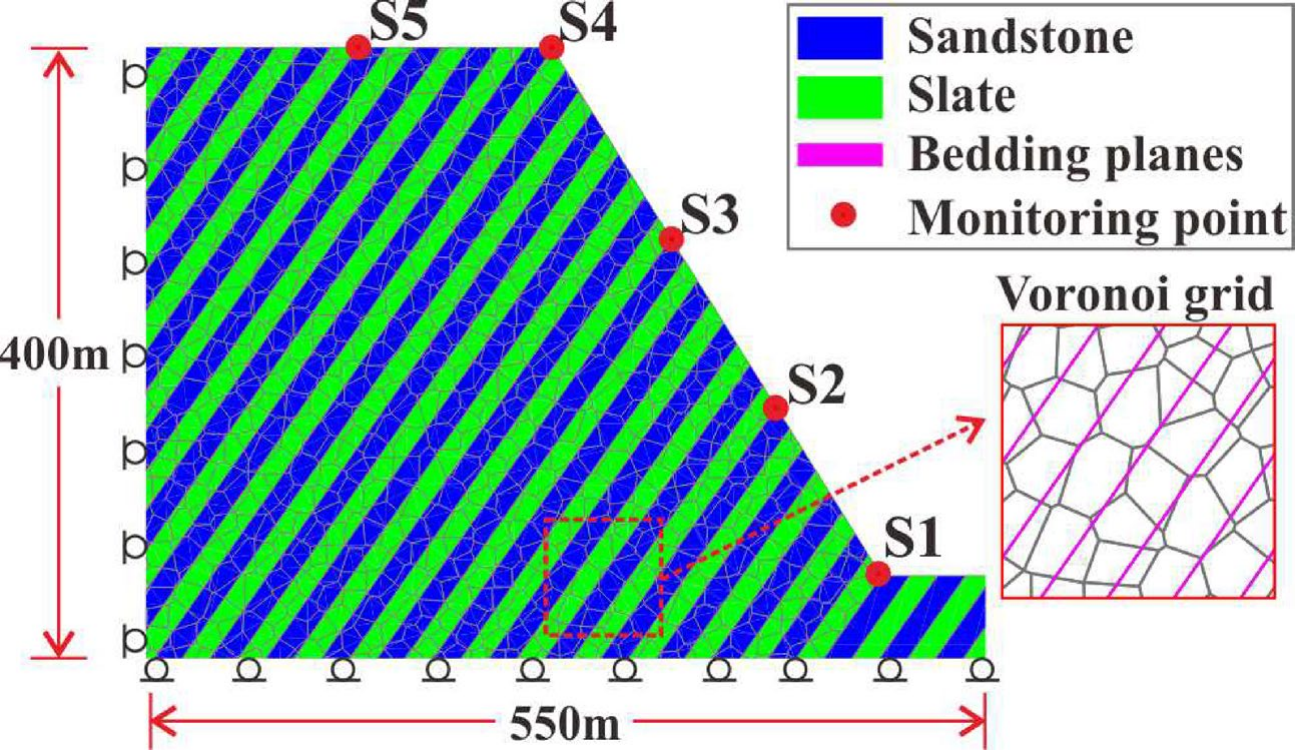
Basic numerical model of the SHIADRS (S1–S5 are monitoring points).

Figure 13 illustrates a basic SHIADRS discrete element model with an SHIR of 1:1 using the UDEC based on the geological profile (Fig. 12). The model dimensions were 550 m in length and 400 m in height, with a slope height of 346.4 m. The field stratigraphic structural facies network statistics (Fig. 8) identified three dominant joint sets (JS1, JS2 and JS3) in the RNL area. In the UDEC model, only the anti-dipping bedding planes (JS1) were explicitly represented as discrete discontinuities. This simplification was justified by field evidence indicating that JS1, due to its high persistence and role as a lithological boundary, constitutes the primary structurally controlled plane of weakness. The less persistent joint sets (JS2 and JS3) contribute predominantly to bulk rock mass strength reduction rather than forming large-scale kinematic release surfaces. Their mechanical effect was implicitly accounted for through the GSI-based scaling of intact rock properties to the rock mass level. Consequently, a single set of bedding planes with a dip angle of 55° was incorporated. Although the field measurements indicated the rock layer thicknesses of 0.8–2.2 m, the thickness was standardized to 15 m in the numerical model to enhance computational efficiency.

To mitigate potential size effect during the crack propagation simulations^40^, the maximum size of the Voronoi-based polygonal blocks within the layers was set to 1.5 m (approximately one-tenth of the layer thickness). This discretization resulted in a total of 1510 discrete block units for the entire model.

The mechanical parameters for the sandstone blocks, slate blocks, and bedding planes were assigned according to the data in Tables 3 and 4. Additionally, five kinematic monitoring points were installed on the slope surface.

#### Construction of different SHIR models

**Fig. 14:**
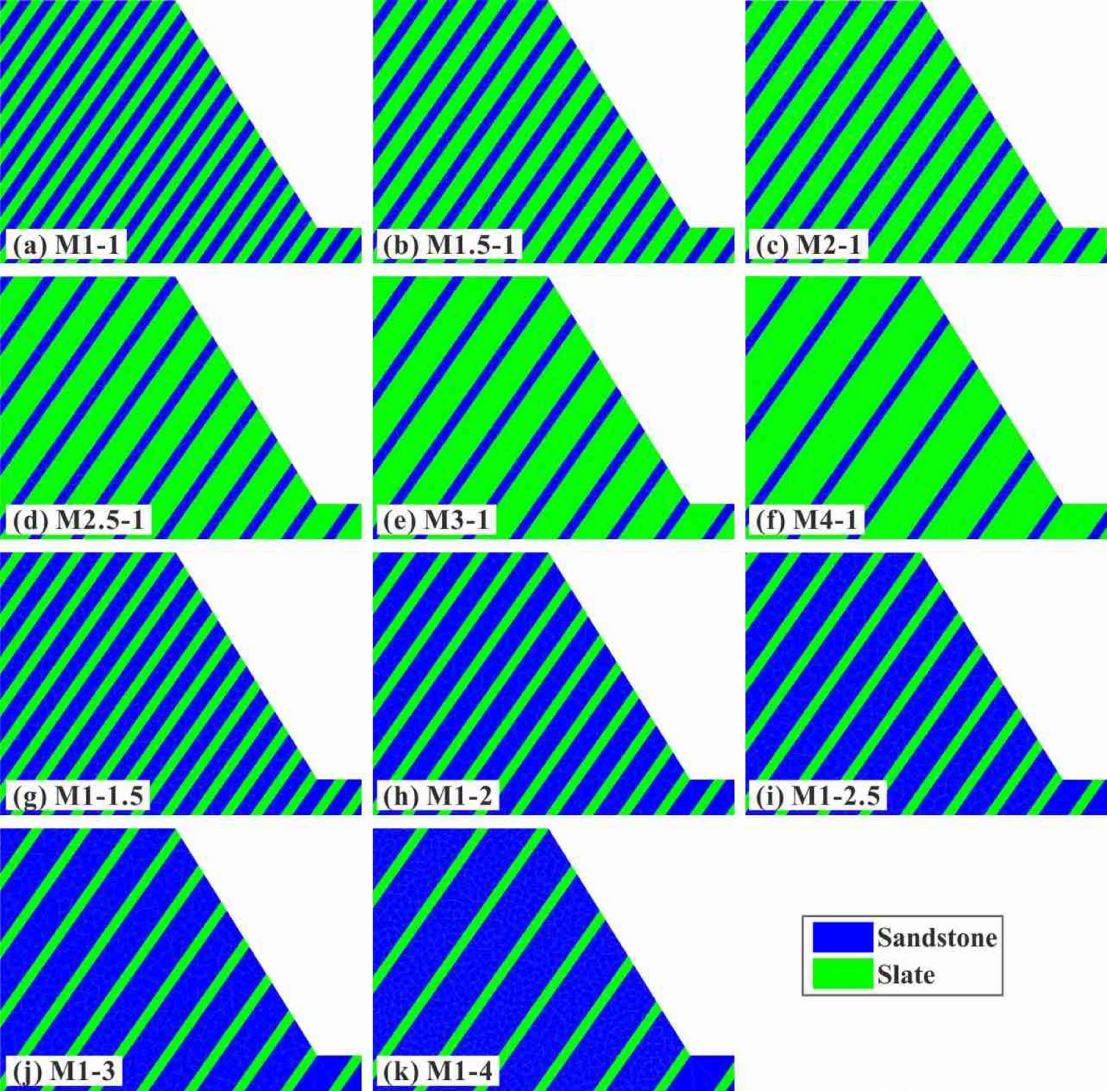
Numerical models of SHIADRS with different SHIRs: (**a**) uniform distribution of soft and hard rock layers; (**b**)–(**f**) soft rock-dominated; and (**g**)–(**k**) hard rock-dominated.

Numerical models of the SHIADRS with eleven different SHIR values were constructed, as presented in Fig. 14. The model set comprised two series: soft rock-dominated and hard rock-dominated. The soft rock-dominated series (Fig. 14b–f) featured a constant hard rock layer thickness of 15.0 m and varying soft rock layer thicknesses of 22.5, 30.0, 37.5, 45.0, and 60.0 m, corresponding to SHIR values of 1.5:1, 2:1, 2.5:1, 3:1, and 4:1, respectively. The hard rock-dominated series (Fig. 14g–k) featured a constant soft rock layer thickness of 15.0 m and varying hard rock layer thicknesses of 22.5, 30.0, 37.5, 45.0, and 60.0 m, corresponding to SHIR values of 1:1.5, 1:2, 1:2.5, 1:3, and 1:4, respectively. For clarity, the models were labeled using the convention M [soft rock thickness ratio]—[hard rock thickness ratio]. For example, M2-1 represents a SHIADRS model with a soft-to-hard rock layer thickness ratio of 2:1.

### Simulation of dynamic failure process

#### Dynamic loading parameters

Based on data obtained from the China Earthquake Administration^41^, $PGA$ in the Zuogong region of Tibet ranged from 0.32 to 0.46 g for rare earthquake (with a 2% probability of exceedance in 50 years). This hazard level defines the design-basis ground motions for the site. The corresponding ${M}_{s}$ varied between 5.0 and 8.0, and $f$ was 2.2 Hz. Applying Eq. (4), the range of ${V}_{max}$ was calculated to be 0.0232–0.0333 m/s. Using Eq. (5), the range of $t$ was calculated to be 0.56–66.07 s. The objective of this back-analysis is to determine the most representative pre-failure SHIR. To avoid the potential bias associated with the extreme values of the parameter ranges, a characteristic seismic input was defined by adopting the average values of $PGA$ and $t$. This approach provides a characteristic seismic input consistent with the defined hazard level, avoiding the potential overestimation or underestimation associated with the extreme ends of the parameter range. Consequently, the following characteristic parameters were selected to induce instability in the RNL models: ${V}_{max}$= 0.0275 m/s and $t$ = 35 s. By substituting these values into Eq. (3), the seismic wave velocity–time history expression for the RNL instability was derived:

13 $$V=0.0275\times \mathit{sin}(13.8\times t).$$

#### Static analysis results

**Fig. 15:**
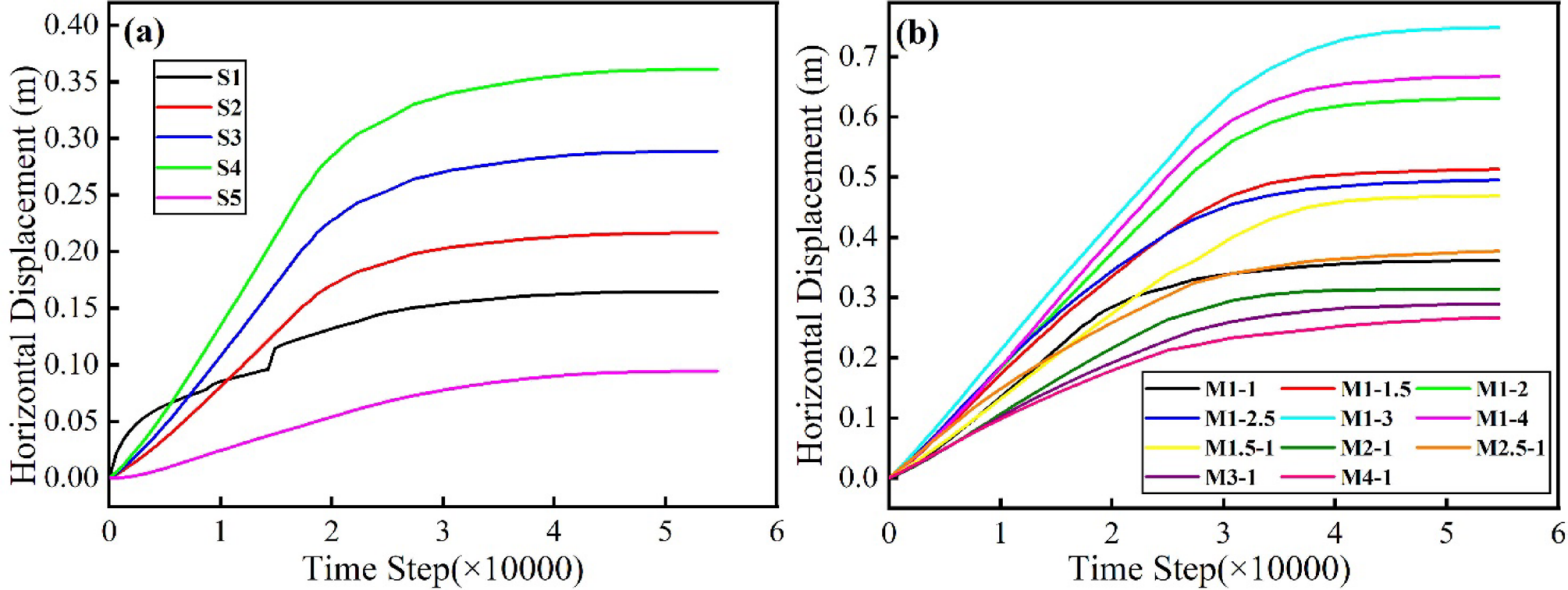
Horizontal displacement time-history curves for the static analysis: (**a**) slope monitoring points in M1-1; and (**b**) monitoring point S4 for all models.

The numerical simulation of slope failure under seismic loading typically comprises two sequential steps: the first step is static analysis, and the second step is dynamic analysis. To ensure the reliability of the subsequent dynamic simulation, it is crucial to verify that the model achieves not only mathematical convergence but also a state of complete physical equilibrium under gravitational loading, thereby precluding any spurious transient creep or plastic flow. The stabilization of horizontal displacement over calculation steps at monitoring points S1 to S5 provides direct and compelling evidence of this equilibrium state. Figure 15a shows the variation of horizontal displacement with calculation steps at these points for model M1-1. Upon completion of the static analysis, all five displacement curves gradually tend toward a horizontal orientation, indicating that horizontal displacements have ceased and the model has reached a state of equilibrium^28^. Figure 15b illustrates the horizontal displacement time histories at the slope crest (S4) for all models. All curves exhibit similar stabilization during the final stages of the static calculation, confirming that every slope model achieved equilibrium prior to dynamic loading. These results collectively demonstrate that, regardless of the SHIR value, the RNL slope remains stable under gravitational loading alone.

Critically, this static phase was designed solely to achieve a stable initial stress field under gravity, without inducing static failure. The resultant equilibrium state, as evidenced by the stabilized displacements in Fig. 15, thereby provides the essential foundation and physically realistic foundation for the subsequent simulation of seismic failure.

#### Dynamic analysis results

**Fig. 16:**
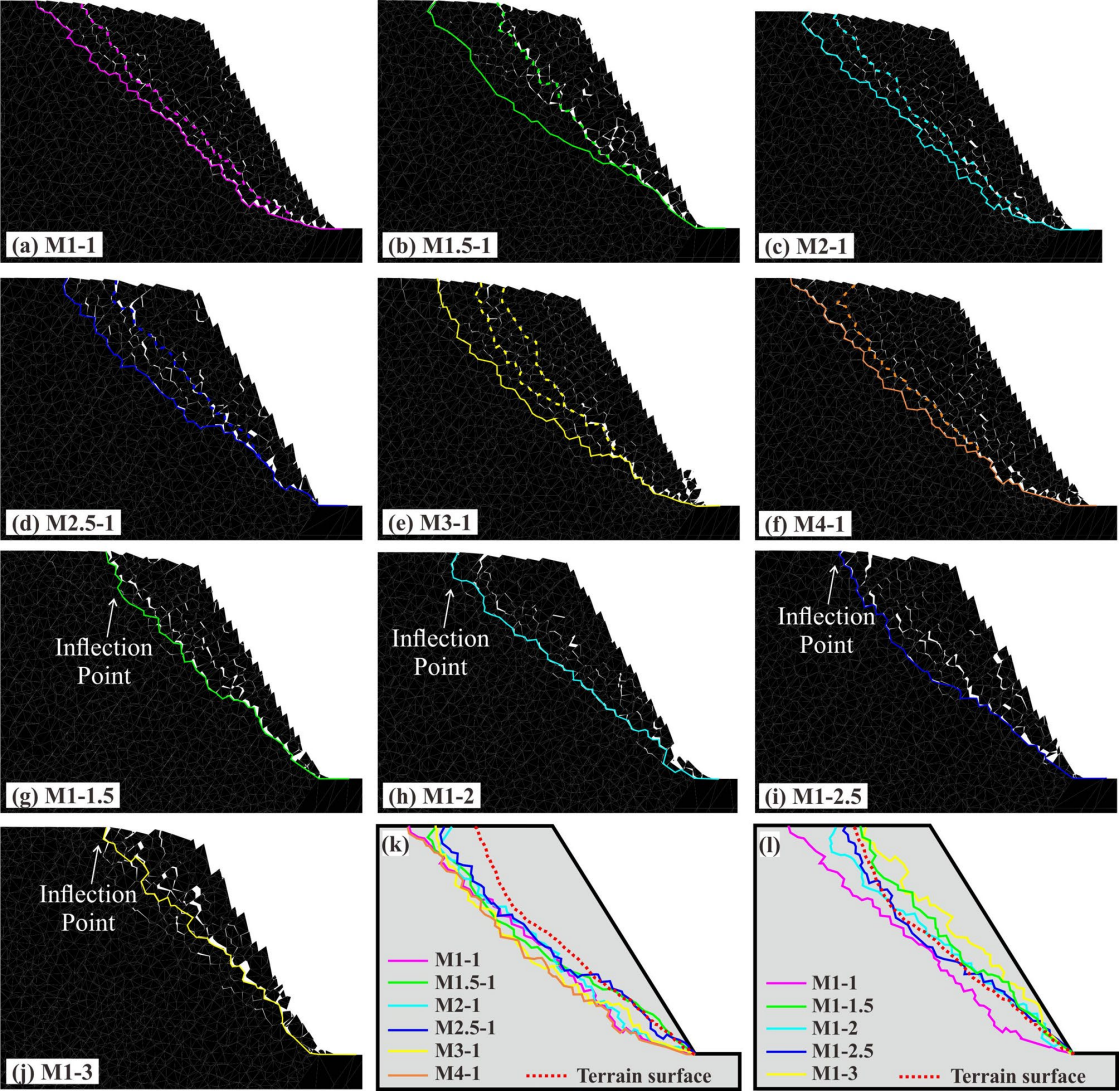
Failure surface morphologies after dynamic instability in various slope models: (**a**)–(**j**) failure surfaces of individual slope models; summarized in (**k**) soft rock-dominated models and (**l**) hard rock-dominated models.

Following the static analysis, seismic loading was applied to each model to simulate the dynamic slope instability. The simulations revealed that under identical dynamic loading parameters, only M4-1 remained stable, while all other SHIADRS models underwent flexural toppling and developed distinct failure surfaces (Fig. 16a–j).

All soft rock-dominated SHIADRS models (SHIR > 1; Fig. 16b–f) developed two failure surfaces, with the deeper one defined as the primary failure surface. The slope toe consistently exhibited a stepped, shear-dominated failure surface. Notably, the geometry of the upper primary failure surface varied with the SHIR: For SHIR = 1:1 and SHIR = 2:1, it formed a relatively continuous, planar segment. For SHIR = 1.5:1, SHIR = 2.5:1 and SHIR = 3:1, it composed of two segments a larger upper concave section and a smaller lower convex section. For SHIR = 4:1, it manifested as a complete arc-shaped concavity. These geometric variations suggest a mesoscopic transition in the failure mechanism from purely brittle bending fracture-toppling to a composite of ductile bending, shear slip, and toppling. A summary of all primary failure surfaces (Fig. 16k) shows that while their morphology is mostly similar, differences are evident in the middle and rear scarp region. In the middle region, the depth of the primary failure surface gradually increased with the SHIR. In contrast, no clear correlation was observed between the SHIR and the failure surface location at the rear scarp. Overall, the failure scale of the SHIADRS tended to increase with increasing SHIR.

All hard rock-dominated SHIADRS models (SHIR < 1; Fig. 16g–j), composite failure surface characterized by a prominent bilinear geometry, with an inflection point near the rear scarp, indicating that brittle fracture prevails in the failure process. As the proportion of the hard rock increased, the number of internal cracks in the upper sliding mass and the bedding shear displacement decreased, demonstrating a gradual transition from ductile flexural toppling to rigid toppling. The summary of all failure surfaces (Fig. 16l) shows distinct differences in the middle region and at the rear scarp. As the SHIR decreased, the failure surfaces moved toward the slope surface in the middle region and toward the free face at the rear scarp. Overall, the failure scale decreased with decreasing SHIR.

Furthermore, a comparison with the actual topographic surface (denoted by a red dotted line in Fig. 16k–l) indicates that the failure surfaces of the hard rock-dominated models exhibit a superior geometric correspondence.

### Determination of the SHIR value

**Fig. 17:**
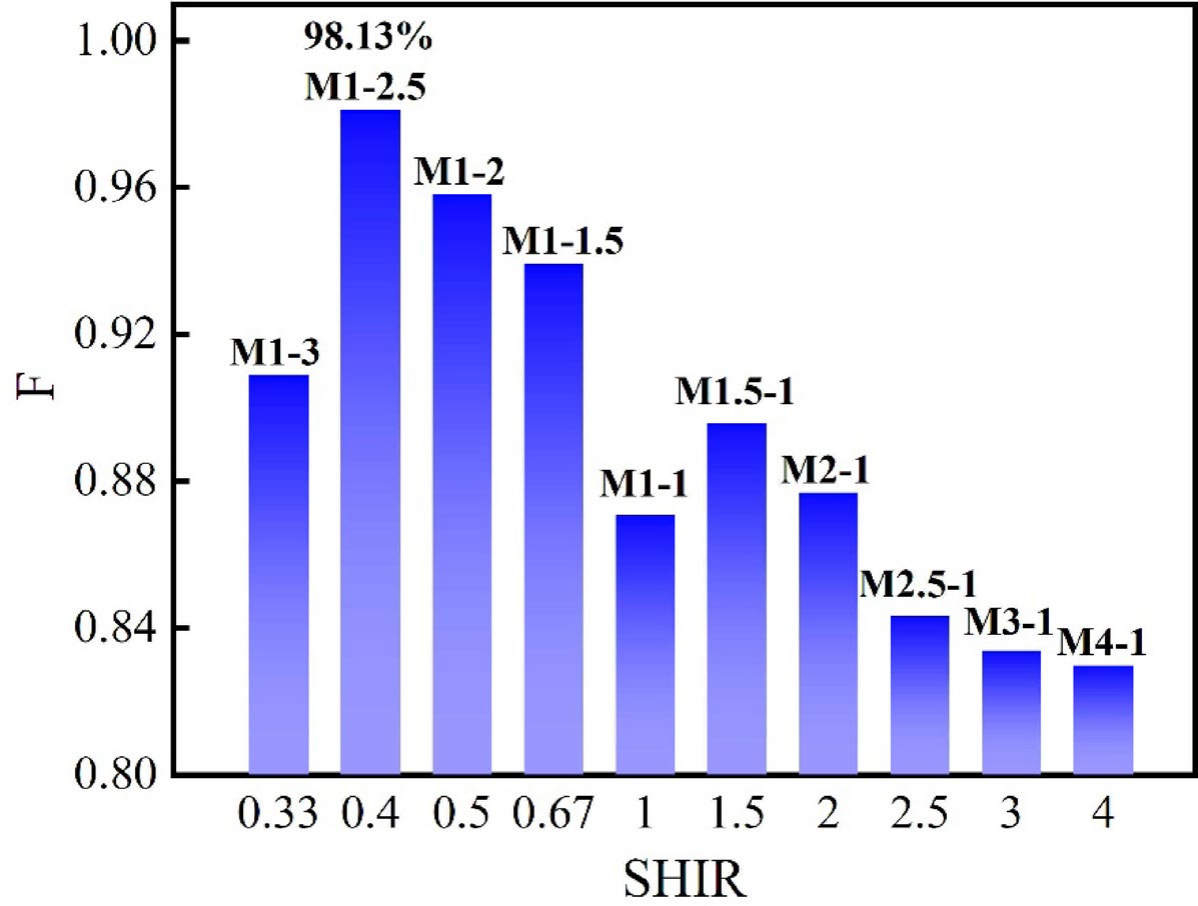
Results of geometric fitness degree calculation for different SHIR values.

Using Eq. (12), the $F$ values between each of 11 simulated failure surfaces and the actual terrain surface were calculated (with a sampling density $N$ of 1000). The results, synthesized in Fig. 17, show that the $F$ value exhibited a distinct nonlinear trend, initially increasing and then decreasing as the SHIR increased from 0.33 to 4. This demonstrate that SHIR exerts a significant control on the geometry of failure surfaces in SHIADRS under seismic loading. A key observation is that for SHIR < 1 (hard-rock-dominated models), the fitness degree consistently maintained a high value, generally exceeding 90%. In contrast, models with SHIR > 1 (soft-rock-dominated) showed markedly lower and more variable $F$ values. This suggests the pre-failure RNL slope was a hard rock-dominated SHIADRS. The optimal SHIR was determined to be 0.4, corresponding to the peak $F$ value of 98.13%. Therefore, the pre-failure stratigraphic structure of the RNL is best represented by a SHIR of 1:2.5 (hard rock-dominated).

## Discussion

### Validation of SHIR back-analysis results

The Toreva blocks observed in the field exhibit the typical geomorphological features of large-scale landslides. Their formation condition can be briefly outlined as follows: during mass movement, the presence of underlying soft rock layers induces a rigid rotation of the overlying rock mass rather than fragmentation. Consequently, these rotated blocks preserve the original stratigraphic assemblage of the slope^42,43^. Five such Toreca blocks were identified in the lower part of the RNL slip source area (Fig. 7a). The thicknesses of the adjacent soft and hard rock layers within these blocks were measurable and, therefore, provided a direct ground-truth benchmark for validating the accuracy of the back-analyzed SHIR.

**Fig. 18:**
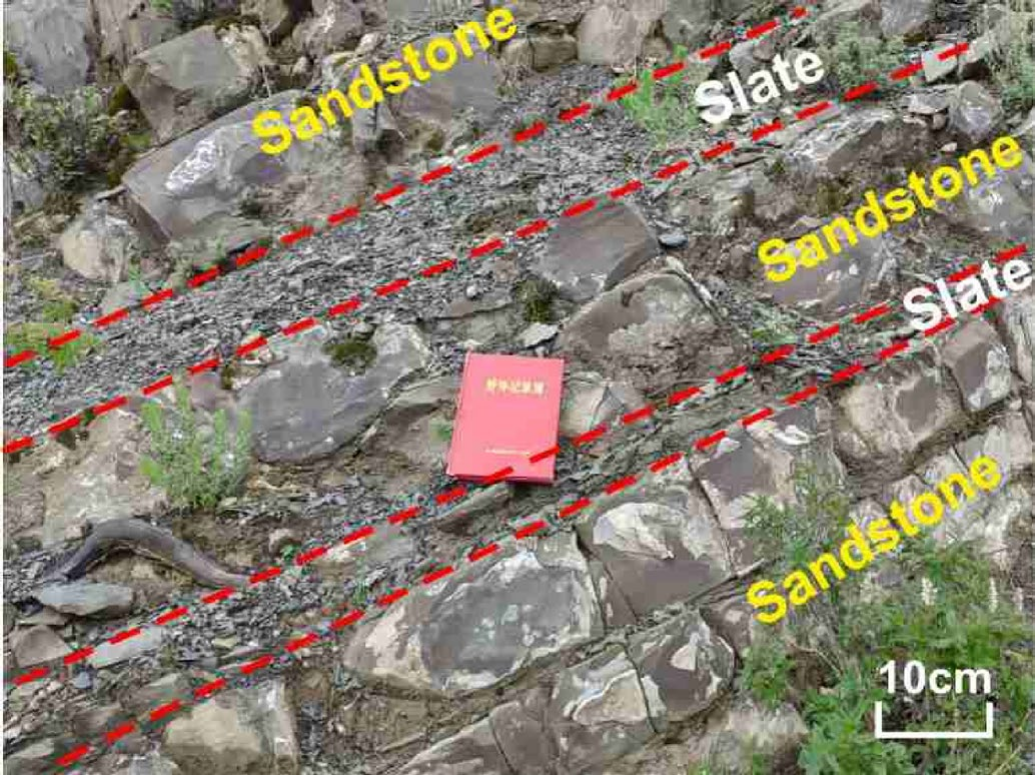
Structural characteristics of alternating hard and soft anti-dip rock layers.

Figure 18 shows the typical interbedded structure of the metamorphic sandstone and slate within one of these Toreca blocks at the bottom of the source area. The metamorphic sandstone appears as coherent blocky, while the slate is more fragmented, with clear lithological boundaries between them.

During the field investigation, eight stratigraphic survey points were established at the edge of the Toreca blocks (for exact locations, see Fig. 7a). At these points, the thickness of adjacent metamorphic sandstone and slate beds were measured. The results are presented in Table 5.

**Table 5:** Information about the stratigraphic structure of the Toreca blocks.

Point No.	Slate thickness (m)	Metamorphic sandstone thickness (m)	SHIR measured value	SHIR simulated value	Absolute error	Relative error (%)
T1	1.61	4.186	1:2.6	1:2.5	-0.1	− 3.85
T2	0.72	1.584	1:1.8		0.3	13.64
T3	0.74	2.294	1:3.1		-0.6	-19.35
T4	0.84	1.596	1:1.7		0.6	31.58
T5	1.01	2.121	1:2.1		0.4	19.05
T6	0.75	2.25	1:3.0		-0.5	-16.67
T7	0.31	0.744	1:2.4		0.1	4.17
T8	1.52	3.496	1:2.2		0.2	8.70
Average value	–	–	1:2.45	–	0.05	4.66

According to Table 5, the close agreement between the back-analyzed SHIR (1:2.5) and the average field-measured SHIR (1:2.45) serves as the primary validation of the proposed methodology. The resulting absolute error is merely 0.05, corresponding to a relative error of 4.66%. These results demonstrate that the proposed back-analysis method can determine the pre-failure stratigraphic structure of SHIADRSs under seismic loading with a high degree of accuracy and reliability.

### Reasonableness of geometry of the failure surface

The accurate simulation of the failure surface constitutes the core of the proposed back-analysis methodology. The numerical results, as quantified by the failure angles in Fig. 16k–l and summarized in Table 6, demonstrate a clear dependency of the failure surface geometry on the SHIR.

**Table 6:** Failure angles of different models.

Model No.	Failure angle (°)	Model No.	Failure angle (°)
M4-1	0 ~ 17	M1-1	0 ~ 5
M3-1	0 ~ 19	M1-1.5	1
M2.5–1	0 ~ 15	M1-2	6
M2-1	0 ~ 5	M1-2.5	8
M1.5–1	0 ~ 16	M1-3	9

A key finding of this study is the divergence of failure modes governed by the SHIR. For slopes with SHIR > 1 (soft-rock-dominated), the failure surfaces are distinctly arc-shaped, exhibiting a wide spectrum of failure angles from 0° to 19°. In contrast, those of slopes with SHIR < 1 (hard-rock-dominated) consistently approximate a linear plane, with narrower range of failure angles between 1° and 9°.

This fundamental distinction between arc-shaped and near-linear failure morphologies aligns with the established body of knowledge from physical modeling. The arc-shaped failures are consistent with the patterns documented in centrifuge tests on soft-hard interbedded anti-dip slopes^11^. Similarly, the transition to near-linear failure planes in our hard-rock-dominated models mirrors the characteristic failure surfaces reported in physical models of single-lithology, hard-rock anti-dip slopes^44,45^.

**Fig. 19:**
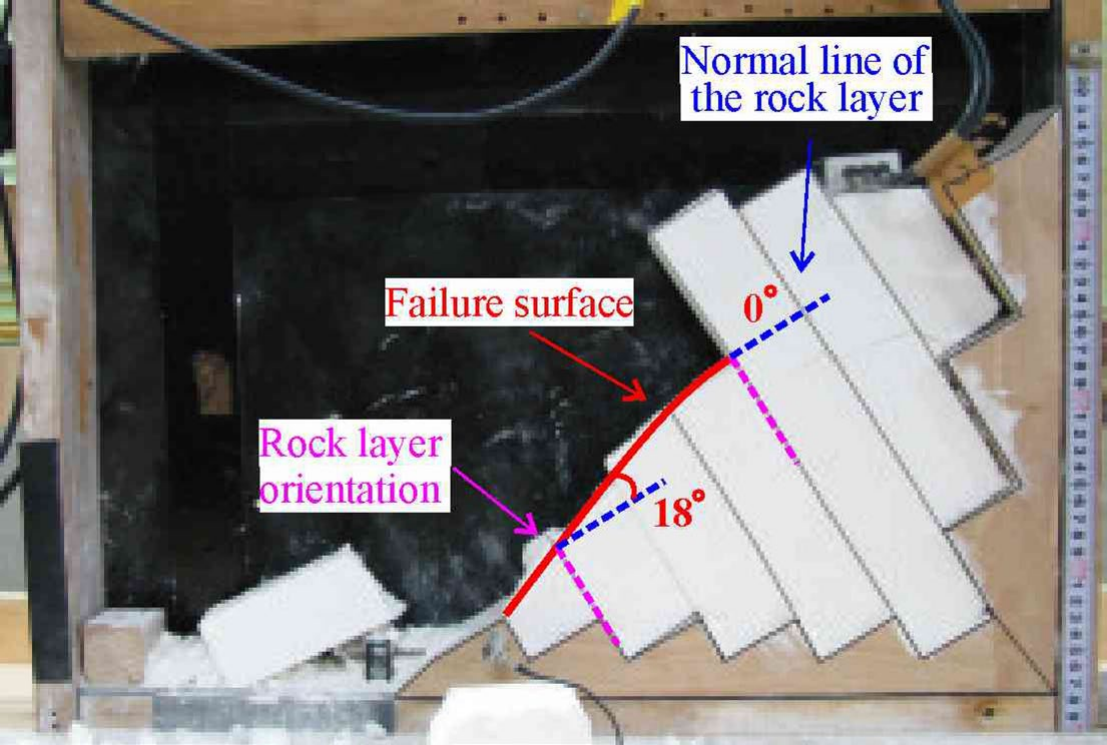
Failure surface characteristics in shaking table tests of anti-dip slopes^46^.

Furthermore, our simulations reveal a significant refinement to this general pattern: the failure angles are generally smaller than those reported in previous static physical models. We posit that this discrepancy is attributable to dynamic-loading effects. This interpretation is strongly supported by the shaking table tests of Aydan et al. ^46^, who also observed reduced failure angles (0°–18°) under seismic conditions compared to static failures (Fig. 19). The consistency between their physical experiments and our numerical simulations under dynamic loading provides robust, cross-validation for our methodology and offers a new mechanistic insight: seismic activity may act to constrict the distribution range of failure angles in anti-dip slopes.

### Model assumptions and limitations

While the proposed methodology effectively back-analyzes the stratigraphic structure of paleolandslides, several assumptions and limitations should be acknowledged.

Firstly, the UDEC models explicitly represented only the dominant bedding planes (JS1), while the influence of less persistent cross-joints (JS2, JS3) was incorporated implicitly through rock mass strength reduction using the Geological Strength Index (GSI). This approach captures the bulk weakening effect but inherently influences the failure mechanism, predisposing the simulation results toward flexural toppling or composite failure modes, as the explicit kinematic freedom for idealized block toppling, which requires well-defined and continuous cross-joints, was not provided. The observed stepped failure surfaces in models with higher SHIR values likely represent a transitional morphology that bridges the spectrum between flexural and block toppling mechanisms.

Secondly, the analysis did not consider the effects of pore water pressure. This assumption is based on field observations from the Rannai landslide site, where the source area shows no evidence of significant groundwater seepage or saturation, suggesting well-drained conditions and a minimal role for pore water pressure in the slope’s final instability. However, it is recognized that for slopes under different hydrogeological conditions, pore water pressure could be a decisive factor and should be incorporated into the analysis.

Lastly, this research focused primarily on the SHIR as the key variable. While SHIR is a fundamental structural parameter, other factors such as the absolute strength contrast between the soft and hard layers, the specific characteristics of the input seismic motion, and the detailed morphology of the pre-failure topography were kept constant or simplified. Future work could explore the sensitivity of the results to these parameters to gain a more comprehensive understanding of the instability mechanisms in such complex slopes.

These limitations, however, do not undermine the primary conclusion regarding the back-analyzed SHIR. Instead, they define the boundary conditions under which our methodology is most applicable and highlight fruitful avenues for future research.

## Conclusions

In this study, we addressed the technical challenge of obtaining complex pre-failure stratigraphic structures of landslides using conventional methods by developing a method that combines high-precision UAV terrain imagery with discrete element numerical simulation to back-analyze the pre-failure SHIR parameter of seismically induced SHIADRSs. The main conclusions of this study are summarized below.

1. Our method encompasses field surveys, high-precision UAV remote sensing, pre-failure topographic reconstruction, construction of SHIADRS discrete element models with varying SHIR values, simulation of the dynamic instability to obtain failure surfaces, and calculation of the geometric fitness degrees using the average horizontal absolute error between the simulated failure surfaces and actual topography. We applied our method to the RNL and determined the optimal SHIR before dynamic instability occurred. Our results show an error of < 5% compared to the field investigation results, validating the feasibility of our method.
2. The UDEC-DM modeling technique successfully simulated the seismic-induced failure process of the SHIADRS and effectively explored how SHIR variations influence the geometric characteristics of the failure surface of the SHIADRS, providing a basis for back-analysis of pre-failure SHIR values.
3. The RNL occurred on a typical SHIADRS. Before dynamic instability and the occurrence of the RNL, the slope was predominantly composed of hard rock. The back-analyzed optimal SHIR was 1:2.5, indicating a shear sliding-flexural toppling failure mode.

This study provides a novel technical approach for reconstructing pre-failure strata structures of SHIADRSs, as well as valuable guidance for rock slope stability evaluation and hazard prediction in seismically active regions.

## Data Availability

All data generated or analyzed during this study are included in this published article.
